# Psychedelics and Immunomodulation: Novel Approaches and Therapeutic Opportunities

**DOI:** 10.3389/fimmu.2015.00358

**Published:** 2015-07-14

**Authors:** Attila Szabo

**Affiliations:** ^1^Department of Immunology, Faculty of Medicine, University of Debrecen, Debrecen, Hungary

**Keywords:** psychedelics, inflammation, autoimmunity, cancer, 5-HTR, sigma-1 receptor, pattern-recognition receptors

## Abstract

Classical psychedelics are psychoactive substances, which, besides their psychopharmacological activity, have also been shown to exert significant modulatory effects on immune responses by altering signaling pathways involved in inflammation, cellular proliferation, and cell survival via activating NF-κB and mitogen-activated protein kinases. Recently, several neurotransmitter receptors involved in the pharmacology of psychedelics, such as serotonin and sigma-1 receptors, have also been shown to play crucial roles in numerous immunological processes. This emerging field also offers promising treatment modalities in the therapy of various diseases including autoimmune and chronic inflammatory conditions, infections, and cancer. However, the scarcity of available review literature renders the topic unclear and obscure, mostly posing psychedelics as illicit drugs of abuse and not as physiologically relevant molecules or as possible agents of future pharmacotherapies. In this paper, the immunomodulatory potential of classical serotonergic psychedelics, including *N*,*N*-dimethyltryptamine (DMT), 5-methoxy-*N*,*N*-dimethyltryptamine (5-MeO-DMT), lysergic acid diethylamide (LSD), 2,5-dimethoxy-4-iodoamphetamine, and 3,4-methylenedioxy-methamphetamine will be discussed from a perspective of molecular immunology and pharmacology. Special attention will be given to the functional interaction of serotonin and sigma-1 receptors and their cross-talk with toll-like and RIG-I-like pattern-recognition receptor-mediated signaling. Furthermore, novel approaches will be suggested feasible for the treatment of diseases with chronic inflammatory etiology and pathology, such as atherosclerosis, rheumatoid arthritis, multiple sclerosis, schizophrenia, depression, and Alzheimer’s disease.

## Introduction

Psychedelics are psychoactive substances that possess the ability to alter cognition and perception by triggering neurotransmitter receptors in the brain. Psychedelics are members of a wider family of psychoactive drugs known as hallucinogens, a class that also includes essentially unrelated psychotropic substances (e.g., dissociatives, deliriants, etc.) (1). These substances affect the mind in unique ways that result in altered states of consciousness, which are qualitatively and phenomenologically different from the ordinary states. According to their pharmacological action, psychedelics usually fall into one of the following categories: *tryptamines*, such as psilocin and *N*,*N*-dimethyltryptamine (DMT); *lysergamides*, most importantly lysergic acid diethylamide (LSD); *phenethylamines*, a large group of diverse substances including 2,5-dimethoxy-4-iodoamphetamine (DOI), and 3,4-methylenedioxy-methamphetamine (MDMA); *cannabinoids*; and *atypical psychedelics*, such as salvinorin A (2, 3). Tryptamines, lysergamides, and phenethylamines are often considered as “classical psychedelics” that exert their effects via the serotonergic system, and a growing body of evidence suggests that they may have therapeutic effects in treating many psychiatric disorders (3, 4).

Scientific investigations concerning the possible immunological effects of psychedelics date back to the early 70s. However, the biomedical Renaissance of psychedelic research has only begun about a decade ago. An important antecedent was the identification of neuro-immune communication in mammals that greatly expanded the domain of physiological activity of psychoactive substances. Since immune cells were found to also express many types of neurotransmitter receptors, an entirely new aspect was added to the biomedical paradigm. Early neuroimmunologists considered the immune and nervous systems as separate parts, but a crucial conceptual leap led to the emergence of the modern approach. This new concept represents neuroimmune communication as an integrated physiological entity with the immune and nervous systems being its two aspects (5, 6).

Many of the naturally occurring psychedelics have been used as a form of traditional medicine by indigenous people since centuries or even millennia (7, 8). These remedies, as inherent parts of the shamanic practice, exert many beneficial effects on the human body (9–11). Unfortunately, the amount of evidence-based, rigorous scientific data about the immunomodulatory functions of psychedelic substances has been quite scarce to date.

In the last two decades, several neurotransmitter receptors involved in the pharmacology of psychedelics have been identified as also being crucial in many immunological processes pointing out to novel therapeutic avenues (12–16). This emerging field offers very promising treatment modalities in the therapy of various diseases including autoimmune and chronic inflammatory conditions, infections, and cancer. However, the paucity of available review literature renders the topic unclear and obscure, mostly posing psychedelics as illicit drugs of abuse and not as possible and effective agents of future pharmacotherapies. In this paper, the immunomodulatory effects of classical serotonergic psychedelics will be discussed from a molecular immunological and pharmacological perspective, and novel approaches will be suggested in the treatment of various pathologies.

## Molecular Basics of Serotonin and Sigma Receptor Signaling

**Figure 1:**
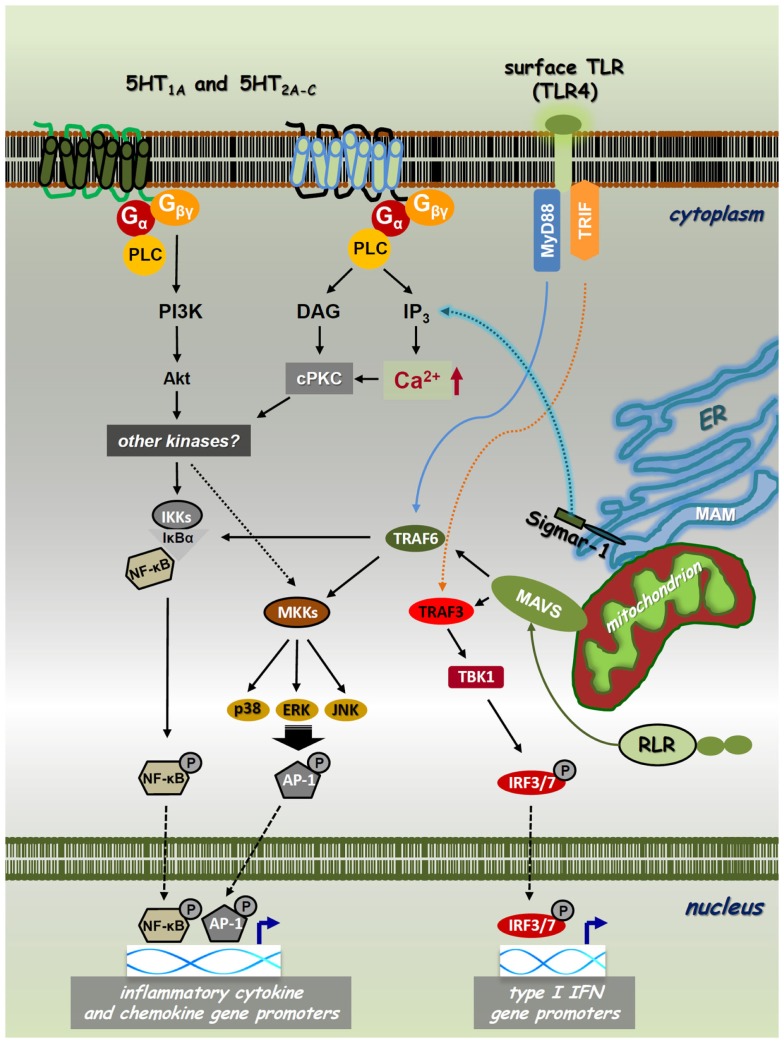
**Cross-talk of PRR, 5-HTR, and sigmar-1 pathways**. Toll-like receptors (TLRs) and RIG-I-like receptors (RLRs) are expressed on the cell surface, localized on intracellular membranes or in the cytoplasm, respectively. These PRRs recognize various sets of pathogenic structures and transduce signals through the NF-κB/IRF pathways. The interaction of a specific PAMP/DAMP with TLRs/RLRs results in downstream signaling through the MyD88/TRIF (TLRs) or MAVS (RLRs) adaptor proteins. This receptor–adaptor interaction leads to the activation of TBK1, MAP-kinase kinases (MKKs), or IKKs via TRAF3 or TRAF6, and leads to the subsequent phosphorylation of IRF3/IRF7, MAPKs-AP-1, or NF-κB, respectively. These transcription factors then translocate to the nucleus regulating the transcription of type I IFN, chemokine, and inflammatory cytokine genes, such as IFNβ, IL-8, IL-1β, IL-6, and TNFα. Classical psychedelics can trigger 5-HT_1A_, 5-HT_2A-C_, and/or sigma-1 receptor (Sigmar-1) signaling and thereby control intracellular Ca^2+^ levels through IP_3_. 5-HTRs and sigmar-1 can use cPKC and Akt to interfere with PRR-mediated NF-κB and MAPK signaling. Thus, NF-κB and MAPK have a cardinal role in both the collaboration and essential signaling processes of PRRs, 5-HTRs, and sigmar-1.

To understand the nature of the psychedelics-immunity cross-talk, we need to briefly discuss the molecular biology of neuro-transmitter receptor pathways involved in the pharmacological actions of psychedelics. Classical psychedelics exhibit agonistic activity mainly at the 5-hydroxytryptamine (5-HT)/serotonin receptor 5-HT_1A_ and 5-HT_2A-C_ classes. These are G-protein-coupled receptors (GPCRs) with analogous biochemical architecture. Their intracellular domains contain sites for phosphorylation for diverse serine–threonine kinases mediating downstream signaling processes. The 5-HT_1A_ subtype primarily signals via G_αi_ proteins activating or inhibiting adenylyl cyclase (AC), phospholipase C (PLC), Src kinase, mitogen-activated protein kinases (MAPKs), and several other effector pathways (17, 18). It also induces the activity of nuclear factor-κB (NF-κB) (19), a transcription factor that controls pro-inflammatory cytokine and chemokine gene expression (Figure 1) (20). The 5-HT_2A_ receptor activates PLC-β leading to the accumulation of inositol phosphates and elevations of intracellular Ca^2+^ in many tissues and cell types (17, 21). It also has the capability to increase Cyclo-oxygenase-2 (COX-2) activity and the release of transforming growth factor beta (TGF-β) via the stimulation of ERK MAPK activity (22, 23). Furthermore, ligated 5-HT_2A_ was shown to interact with the Janus kinase (Jak)/signal transducers and activators of transcription (STAT) pathway controlling a rapid tyrosine phosphorylation of Jak2 and STAT3 that leads to the nuclear translocation of STAT3 (24). The human 5-HT_2B_ receptor has 45% structural homology with the 5-HT_2A_ and 42% homology with the 5-HT_2C_ subtypes (25). The functional 5-HT_2B_ protein is widely expressed not only in the brain but also in many peripheral tissues. In early studies, it has been showed that 5-HT_2B_ receptors can activate the Ras and ERK1/ERK2 MAPKs involving G_αq_, G_αi_, and G_βγ_ protein activities, and thereby modulate cellular proliferation and differentiation (26). Similarly to 5-HT_2A_ and 5-HT_2C_ receptors, the 5-HT_2B_ receptor couples to the PLC-inositol 1,4,5-trisphosphate (IP_3_) system directly controlling the release of Ca^2+^ from intracellular stores (Figure 1) (17). The ligation of 5-HT_1_ and 5-HT_2_ receptors can directly alter cellular functions in immune cells. In an important study, 5-HT_1_ and 5-HT_2_ receptor stimulation was shown to induce intracellular Ca^2+^ mobilization via G_αi_ proteins in resting, but not lipopolysaccharide (LPS) activated DCs (27). The 5-HT_2C_ receptor has also been shown to modulate PLC-IP_3_ activity (28), and has recently been described as indispensable for the serotonin-mediated activation of murine alveolar macrophages (29). Interestingly, the 5-HT_1_ and 5-HT_2_ receptors have a high expression profile in mammalian lymphoid tissues and involved in many immunological processes (30–32). These include anti-tumor and anti-viral immune responses (31, 33, 34), and the neuroendocrine regulation of inflammation via serotonin as a key factor in immune homeostasis (15, 35, 36).

The sigma-1 receptor (Sig-1R or sigmar-1) is a small integral membrane protein consisting of a short N-terminus, a large C-terminus tail, and two transmembrane domains (37, 38). Sigmar-1 localizes at the endoplasmic reticulum (ER)–mitochondrion interface, also called mitochondria-associated ER membrane (MAM). Previous studies have shown that sigmar-1 interacts with numerous cellular components, such as GPCRs and ion channels (e.g., Na^+^, K^+^, and Ca^2+^). Importantly, similar to 5-HT receptors (5-HTRs), sigmar-1 can also enhance or block the activity of Ca^2+^ channels and thereby regulate intracellular Ca^2+^ levels (38–40). Recently, DMT has been identified as a natural, endogenous ligand for sigmar-1 (41). Ligation by DMT causes the dissociation of sigmar-1 from binding immunoglobulin protein (BiP), allowing it to act as a molecular chaperone to IP_3_ receptors (42). This activation leads to enhanced Ca^2+^ signaling and a significant increase in the production of adenosine triphosphate (Figure 1) (43). Although it resides primarily at the ER, sigmar-1 directly translocates from the MAM to the plasma membrane or the subplasma membrane area following its activation by higher concentrations of specific ligands or when the receptor is overexpressed in cells (44–46). This may explain why the concentration of DMT-modulating cellular physiology is almost 10-fold as compared to its affinity concentration (41, 42). Early studies demonstrated that sigmar-1 is expressed not only in distinct regions of the CNS but also in immune cells (47–49). Murine studies also showed that the specific activation of sigmar-1 resulted in immunosuppression (50), and *in vivo* decreased lymphocyte activation and proliferation (51). Sigma-1 receptor ligands possess potent immunoregulatory properties via increasing the secretion level of anti-inflammatory IL-10 (52), and suppressing interferon (IFN)γ and GM-CSF expression (51).

## Innate Immune Recognition and the Biology of Inflammation and Interferon Responses

**Figure 2:**
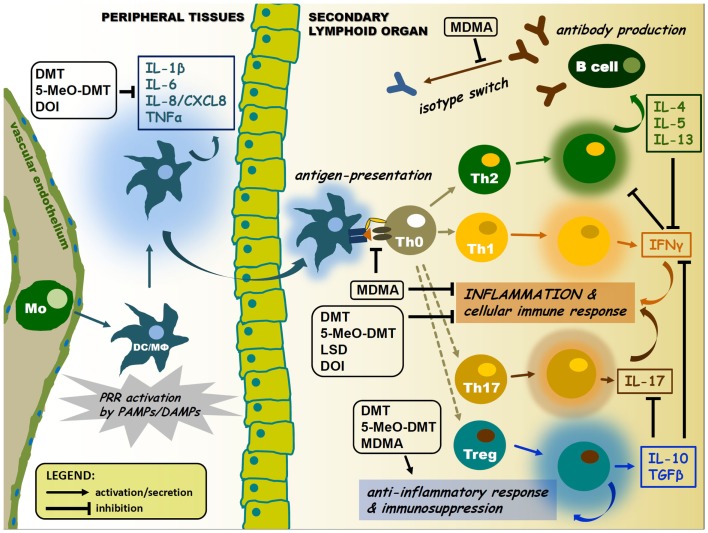
**Pharmacological modulation of APC and lymphocyte cytokine signaling by psychedelics**. Psychedelics can significantly interfere with immune cell cytokine profiles. This may lead to suppression of antigen presentation and inflammatory cytokine and chemokine secretion, as well as inhibition of isotype switching or elevated levels of anti-inflammatory cytokines in the tissue environment. Arrows represent activation or migration of cells, or secretion of cytokines. T-arrows mean inhibition. Abbreviations: Mo, monocyte; DC, dendritic cell; MΦ, macrophage; colored halos around cells represent activation/cytokine secretion.

The immune system acts as an evolutionally conserved and advanced host defense mechanism against invading pathogens. Innate immune responses are triggered by phylogenetically conserved microbial components that are essential for the survival of a given type of organism. Upon pathogenic infection, these pathogen-associated molecular patterns (PAMPs) are recognized by specific pattern-recognition receptors (PRRs) that are germline encoded and are usually expressed constitutively in the host (53–55). The overall picture, however, is far more complex as successful microbial moieties are also found in non-pathogenic microbes, and thus the presence of different PAMPs *per se* is not sufficient to discriminate “pathogenic” and “non-pathogenic” microbial taxa. Furthermore, certain PRRs also sense host-derived/“self” components that become available as a result of cellular/tissue injury. The list of these endogenous damage-associated molecular patterns (DAMPs) is continuously growing but their impact on immune homeostasis is yet to be clarified (Figures 1 and 2) (20, 56). Thus far, five classes of PRRs have been identified. Two important classes are: (i) transmembrane toll-like receptors (TLRs), which are integrated to cell surface or endosomal membranes of various cell types; (ii) cytosolic RIG-I-like receptors (RLRs) (57–59). Upon binding of their specific ligands, these PRRs activate the NF-κB and the IFN-regulatory factor 3/7 (IRF3/7) pathways, as well as MAPKs, such as p38, ERK1/2, and c-Jun N-terminal kinase (JNK) (60, 61). This process altogether results in the expression of a common set of genes whose products, such as inflammatory cytokines, chemokines, and co-stimulatory molecules, are essential for the orchestration of both innate and adaptive immunity (Figure 1). TLR and RLR ligation results in the activation of myeloid differentiation primary response gene 88 (MyD88) or the TIR-domain-containing adapter-inducing IFN-β (TRIF) adapter proteins for TLR pathways, and the mitochondrial adapter mitochondrial anti-viral-signaling protein (MAVS) that mediates RLR downstream signaling (62). TRIF and MAVS then couple to the TNF receptor-associated factor 3 (TRAF3) conveying the signal to TANK-binding kinase 1 (TBK1) through TRAF family-member-associated NF-κB activator (TANK) binding (63). Activated TBK1 induces the phosphorylation of IRF3/IRF7 on specific serine residues, resulting in their homodimerization (64). These dimers then translocate to the nucleus inducing the transcription of type I IFN genes, a cytokine family that is highly involved in anti-viral and anti-tumor immunity (Figure 1) (65). This pathway is implicated to be connected to the NF-κB activation pathway through the interaction of FAS-associated via death domain (FADD), Receptor-interacting protein (RIP1) and TRAF6, which result in the induction of pro-inflammatory cytokine genes and proteins, such as IL-1β, IL-6, and TNF-α (66). The activation of these pathways are crucial in anti-pathogenic immune responses, but are also involved in autoinflammatory and autoimmune pathologies where undesirable inflammation causes chronic and severe damage to self tissues (67).

## Molecular Mechanics of Interacting PRR, Serotonin, and Sigma-1 Receptor Pathways

Many of the classical psychedelics have the capability to interfere with both innate and adaptive immunity. This modulatory potential is usually manifested through the inhibition of inflammatory responses and antigen presentation, and specific, disparate regulation of the proliferation and function of certain lymphocyte subtypes, such as cytotoxic T-lymphocytes (CTLs) or NK cells. The receptors involved in the pharmacology of classical psychedelics are mainly expressed by neuronal cells, and their function in the CNS is well described. However, they are also expressed by immune and hematopoietic cells, and the details of their modulatory potential have not been elucidated yet (68). Regrettably, we have a very limited understanding of these neuroimmune signaling events thus far.

The cross-talk between immune sensors and receptors involved in the pharmacology of psychedelics may occur at multiple levels. Two possible ways of this communication will be proposed. First, an inter-cellular interaction may be established by means of cytokine regulation among various immune cell and tissue types. The classical psychedelics discussed in this paper are acting at either one or all of the 5-HT_1A_, 5-HT_2A_, 5-HT_2B_, and 5-HT_2C_ serotonin receptor subtypes. The activation of these 5-HTR subtypes displays an unique effect on the production of cytokines, which has similar immunological functions, such as IL-1β and TNFα (69). 5-HT receptor activation results in a decrease of TNFα, but an increase in IL-1β secretion in human peripheral blood mononuclear cells (PBMCs) (70), DCs (27), and monocytes stimulated with PRR ligands (71). Furthermore, serotonin was shown to facilitate the production of the pro-inflammatory IL-16 and IFNγ by activated CTLs and NK cells (72). Thus 5-HT receptor agonism appears to control the inflammatory response by regulating different patterns of cytokine secretion (69). Additionally, another key factor here is the negative feedback regulation of inflammation via the induction of the release of anti-inflammatory IL-10 and TGFβ occurring subsequent of 5-HT_1_ and 5-HT_2_ receptor activation (Figure 2) (73–75).

Second, 5-HTR activation, besides its influence on the complex cytokine-feedback regulation, may also interfere with the chemokine, inflammatory cytokine, and/or type I IFN receptor signaling of immune cells through intracellular mechanisms. Most of the receptors that are involved in psychedelic effects belong to the GPCR family or interact with GPCRs (e.g., sigmar-1) (68). The role of 5-HTR/GPCR-coupled signals in the intracellular regulation and orchestration of NF-κB, type I IFN, and MAPK pathways may be of particular importance regarding the complex immunological effects of psychedelics. GPCR agonists have already been described as potent inducers of cytokines, adhesion molecules, and growth factors [reviewed in Ref. (76)]. Specific stimulation of the 5-HT_1_ and 5-HT_2_ receptor subtypes leads to the activation of NF-κB and several MAPKs in many cell types including immune cells (77–81). This 5-HTR-mediated, coordinated cross-talk between MAPKs (including p38, MEKK1, ERK, and PI3K/Akt) and NF-κB leads to an intricate fine-tuning of inflammatory responses by the spatio-temporal regulation of cyokine release. The inhibitory or stimulatory effect of GPCR activation on NF-κB and MAPK pathway kinetics is largely depending on the G-proteins that are involved. Psychedelics, acting through mainly 5-HT_1_ and 5-HT_2_ receptors subtypes, regulate NF-κB and MAPKs via G_α_ (G_i_ and G_q_ families), and G_βγ_ proteins (17–19, 26). The G_q_ family of α subunits couples a large number of GPCRs to PLC-β, and many of these have been shown to activate NF-κB. This mechanism is based on the activity of IκBα and the IκB kinases (IKKs), IKKα and IKKβ, as well as the phosphatidylinositol 3-kinase (PI3K) pathway involving the serine/threonine protein kinase Akt (82). The PLC-β-IP3 axis-mediated release of Ca^2+^ from intracellular stores results in the activation of the second messenger conventional protein kinase C (cPKC) (Figure 1). As mentioned above, this calcium signal can also be attenuated by the activation of sigmar-1 (42, 43), and it is tempting to speculate that sigmar-1 may couple to MAPK and NF-κB signaling and regulate inflammation through this mechanism as well. Several PKC isoforms are known to activate NF-κB, consequently, the G_q_-mediated activation of NF-κB is the result of PLC-β-controlled convergence of IKK and cPKC signaling (76). The G_i_ proteins do not activate PLC-β, but use the G_βγ_ class to signal through MAPKs and induce NF-κB phosphorylation and nuclear translocation (83, 84). Following GPCR activation, G_βγ_ dissociates from G_α_ and can *per se* stimulate both PLC-β and PI3K. This allows a direct control of NF-κB transcriptional regulation of chemokines, pro-inflammatory, and anti-inflammatory cytokines, and thus rendering psychedelics as potentially useful therapeutic tools in a broad range of chronic inflammatory and autoimmune diseases (85).

Another possible mechanism has been raised by recent meta-analyses showing that serotonin signaling could prevent the type I IFN-mediated depressive behavior of HCV patients (86, 87). The signaling behind this phenomenon has not been uncovered yet; however, it is possible that chronic 5-HTR stimulation may block either the PRR-IRF3/7 or type I IFN receptor pathways. Since both NF-κB and type I IFN signaling contribute to the transcriptional regulation of genes that are involved in cellular proliferation and survival, and many psychedelics exhibit *in vitro* anti-cancer potential through 5-HTRs, these compounds could be promising candidates in novel therapies of cancer (88–90).

## Tryptamines: Endogenous Regulators of Inflammation and Tumor Immunity?

Tryptamines are members of a large family of monoamine alkaloids that are widespread in nature and abundant in all the three Kingdoms of life (plants, fungi, and animals). Their main feature is a common indole ring, a backbone that is structurally related to the amino acid tryptophan. This tryptamine backbone designates many biologically active compounds, such as psychedelics and neurotransmitters (91). To date, our knowledge about the immunomodulatory capacity of tryptamines is quite scarce. DMT is the only member of the family that has been investigated so far.

*N*,*N*-dimethyltryptamine is related to the neurotransmitter serotonin, the hormone melatonin, and other psychedelic tryptamines, such as bufotenin and psilocin. It is a naturally occurring indole alkaloid that is ubiquitous in plants, such as *Diplopterys cabrerana* and *Psychotria viridis*, which are used for the preparation of sacramental psychoactive brews including *yage* and *ayahuasca* (92). In addition to its ubiquitous presence in plant species, DMT has also been detected in animal tissues and is considered to be an endogenous trace amine (93). The milestones of DMT research were laid down by Szara (94) and Axelrod (95) who reported first the psychoactive effects and occurrence of this compound in the human brain. This led to the hypothesis that DMT is an endogenous hallucinogen (96, 97), and later it was proposed to be a neurotransmitter or neuromodulator (98). DMT was shown to act as an agonist at several serotonin receptors including 5-HT_1A_, 5-HT_2A_, and 5-HT_2C_ (99–102) as well as at sigmar-1 (41).

The vast majority of the initial research into the reasons for the presence of psychoactive tryptamines in the human body has sought their involvement in mental illness. Until now, very little has been known about the function of DMT in cellular and general physiological processes, and the emphasis of research mostly aimed the understanding of its psychedelic properties (103). Recently, we and others demonstrated that DMT has the capability to modulate immune responses in *in vitro* human primary cell cultures (88, 104). In these studies, DMT was shown to act as a non-competitive inhibitor of indoleamine 2,3-dioxygenase (IDO) and as a strong inducer of anti-tumor cytotoxic activity in the co-cultures of human PBMCs and a glioma cell line (88). Furthermore, DMT and its analog 5-methoxy-*N*,*N*-dimethyltryptamine (5-MeO-DMT) were found to exert potent anti-inflammatory activity through the sigmar-1 in human monocyte-derived dendritic cell (moDC) cultures. MoDCs are key cell types of the mammalian immune system connecting and orchestrating innate and adaptive immune responses as professional antigen-presenting cells (APCs) (20). DMT or 5-MeO-DMT treatment of LPS, polyI:C or pathogen-activated human primary moDCs resulted in a significant inhibition of the secretion of the inflammatory cytokines, IL-1β, IL-6, TNFα, and the chemokine CXCL8/IL-8. In contrast, secreted levels of the anti-inflammatory IL-10 increased markedly following *in vitro* DMT/5-MeO-DMT administration. DMT and 5-MeO-DMT exhibited the effective inhibitory potential at the level of adaptive immune responses (T helper cell 1 and 17 priming by moDCs), as well (104). These are in line with previous findings showing the immunomodulatory potential of ayahuasca in humans mostly affecting the number and ratio of lymphocyte subpopulations. Notably, the number of circulating NK cells, a cell type involved in anti-viral and anti-cancer immune responses, increased significantly (105, 106). The anti-cancer activity of ayahuasca has already been reviewed in a paper by Schenberg (89). However, it is important to keep in mind that ayahuasca is a complex decoction that, besides DMT, contains several other components according to the admixture plants used in the making process. Furthermore, ayahuasca can be administered in various ways (single-time, long-term, etc.), thus one should be particularly careful with the study design and interpretation of the data. Nevertheless, ayahuasca consumption in a highly controlled clinical setting emerges as a very promising model for investigating the possible immunomodulatory effects of DMT in humans (107). Importantly, it is possible that the observed anti-inflammatory and immunosuppressive effects may counteract with the anti-cancer activity, therefore further investigations are needed to elucidate the complex *in vivo* consequences of DMT administration.

The mentioned studies demonstrate and propose new biological roles for DMT, which may act as a systemic endogenous regulator of inflammation and immune homeostasis. According to these new results, DMT and 5-MeO-DMT possess the capability to inhibit the polarization of human moDC-primed CD4^+^ T helper cells toward the inflammatory Th1 and Th17 effector subtypes in inflammatory settings. This is of particular importance, since Th1 and Th17 cells and the cytokines they secrete are key players in the etiology and symptomatology of many chronic inflammatory and autoimmune diseases of the CNS and other tissues (108, 109). Moreover, the mobilization of innate immune mechanisms is also well established in many psychiatric and neurological disorders (6). Thus, as a target for future pharmacological investigations, DMT emerges as a potent and promising candidate in novel therapies of peripheral and CNS autoimmune diseases (such as multiple sclerosis or amyotrophic lateral sclerosis) and cancer.

## Lysergamides: Modulating Lymphocyte Functions

Lysergic acid diethylamide (also know as LSD-25 or lysergide) is a psychedelic substance of the ergoline family. Its pharmacological effects are very complex as it affects several serotonin, as well as all dopamine and adrenoreceptor subtypes. Since most serotonergic psychedelics do not exhibit dopaminergic activity, LSD is quite unique in this regard (110). In humans, LSD mostly affects the 5-HT_1A_, 5-HT_2A_, 5-HT_2B_, and 5-HT_2C_ serotonin receptor subtypes (111). Furthermore, LSD has a functional selectivity at the 5-HT_2A_ and 5-HT_2C_ receptors by specifically activating PLA2 but not PLC (112).

An early study demonstrated that LSD was able to interfere with antibody production in rabbit (113). In this report, LSD was shown to skew the antibody profile of activated B cells to produce low molecular weight proteins by influencing the process of translation. Excess tryptophan abrogated the effect of LSD on protein synthesis suggesting that the phenomenon may occur at the point of tryptophan insertion during translation. However, the data provided did not adequately support a peptide termination mechanism rather reflected an amino acid analog effect being simulated by LSD (113). These results were in line with the findings of another group showing that *in vitro* exposure to high LSD concentration (100 μM) could significantly inhibit the proliferation and IL-2, IL-4, and IL-6 secretion of B cells, as well as blocked CD8^+^ CTL activation (90). Hundred micromoles of LSD also suppressed NK cell responses *in vitro*; however, inversely, lower concentrations of LSD (0.0001 and 0.1 μM) augmented NK cell functions (90). This latter, low concentration can easily be achieved by recreational doses of LSD in humans (111), and therefore may have a significant impact on *in vivo* anti-tumor and anti-viral immune responses. Human lymphocytes express the 5-HT_1A_, 5-HT_2A_, 5-HT_2B_, and 5-HT_2C_ subtypes suggesting that LSD may directly modulate cellular functions through these receptors (114, 115). The results obtained so far suggest that LSD may interfere with the elements of the immune system by altering mainly the activity of lymphocytes in mammals. High doses of this substance may alleviate or inhibit adaptive autoimmune responses, while lower doses may positively influence the anti-viral or anti-cancer immunity through the modulation of NK cell activation. However, detailed analyses on the complex *in vivo* effects of LSD on immune functions are yet to be performed.

## Phenethylamines: Regulating Inflammation and Cytotoxicity

Phenethylamines (or substituted phenethylamines) are members of a large and diverse group of organic compounds, which derive from *phenethylamine* itself. Some of them are neurotransmitters, such as dopamine and epinephrine, other members of the family are psychoactive substances (e.g., entactogens or psychedelics), which directly modulate the monoamine neurotransmitter systems, such as the substituted amphetamines, the substituted methylenedioxyphenethylamines, and several other naturally occurring alkaloids (116, 117). This large family also includes a variety of drug classes, such as dopamine agents (e.g., bupropion), serotonin agents (e.g., the psychedelic 2,5-dimethoxy-4-bromoamphetamine), adrenergic agents (e.g., the adrenergic uptake inhibitor methamphetamine), and monoamine oxidase inhibitors (MAOIs) (118).

Considering the vast number and diversity of substituted phenethylamines, a comprehensive review about the complex immunological effects of these compounds would exceed the limits of this paper. Therefore, this section focuses on two phenethylamines, DOI and MDMA, which have already been described as potential immunomodulators in higher vertebrate species. These psychedelics have several similarities in their pharmacological action as both of them exhibit a certain degree of agonistic activity at serotonin receptors. DOI acts as a 5-HT_2A_, 5-HT_2B_, 5-HT_2C_, and mGlu2 receptor agoinst (119, 120), while MDMA is primarily a presynaptic releasing agent of serotonin, norepinephrine, and dopamine, but also a weak-to-medium agonist at 5-HT_1_ and 5-HT_2_ receptor subtypes (121–123).

Dimethoxy-4-iodoamphetamine was originally designed and used as a radioligand for the mapping of 5-HT_2_ receptors (124, 125). As a 5-HT_2A_ agonist, DOI was reported to block IL-1β and TNFα release by human PBMCs (70) as well as to inhibit LPS or TNFα-stimulated inducible nitric oxide synthase (iNOS) activity in C6 glioma cells (126, 127). In a landmark paper, the Nichols lab described DOI as an extremely potent inhibitor of TNFα-induced inflammation in rat’s primary aortic smooth muscle cell cultures (128). In this report, DOI was shown to inhibit the constitutively high protein-level expression of intracellular adhesion molecule 1 (ICAM-1), vascular adhesion molecule 1 (VCAM-1), the inflammatory cytokine IL-6, and the activity of NOS, as well as NF-κB signaling. DOI exhibited a fast and effective blocking capacity on the expression and function of TNFα-mediated pro-inflammatory markers within a few hours post-administration suggesting that DOI could be used not only to prevent inflammation but also to treat already ongoing inflammatory tissue damage, such as in allergic asthma (128, 129).

The most researched phenethylamine, MDMA, has also been described as an anti-inflammatory and immunosuppressive agent. Early studies reported that MDMA could increase the activity of mouse NK and T helper cells in *in vitro* cultures at low concentrations (0.0001–1.0 μM). The TNFα production of macrophages and the induction of CTLs were suppressed upon MDMA administration (130). Acute administration of MDMA led to significant immunosuppression by directly decreasing lymphocyte proliferation and blocking the mitogen or LPS-induced cytokine (IL-1β and TNFα) production of T cells in *in vivo* animal models (131–133). Later, IL-10 was shown to be the critical mediator in these immunosuppressive effects on IL-12 and IFNγ production in mice (134). MDMA has also been demonstrated to interfere with isotype switching blocking the conversion of IgM to IgG2a (135), as well as decreasing the expression of MHC-II and the co-stimulatory molecules, CD40, CD80, ICAM-1 suppressing the T cell-priming capacity of professional APCs (134). Furthermore, several groups reported that MDMA negatively affected *in vivo* immune responses to various pathogens in animal models (132, 136–140). In agreement with these findings, both acute and chronic MDMA administrations were demonstrated to cause immunosuppression in humans characterized by a significant decrease in T lymphocyte and parallel increase in NK cell functions. Long-term use of MDMA, however, was associated with a decrease in the total number of circulating lymphocyte populations. These alterations also involved a significant decrease in the plasma level of IL-2 and increase of TGF-β in human volunteers (141–144). These results suggest that acute administration of MDMA favors anti-inflammatory immune responses and has a tendency to polarize adaptive immunity toward antibody production. Simultaneously, the activity of NK cells is increased pointing to a complex effect on immune homeostasis. This may reflect to an anti-inflammatory potential of MDMA without significantly decreasing the effectiveness of anti-viral or anti-tumor immunity; however, further *in vivo* studies are needed to unravel the details of this complex immunomodulatory action.

## Discussion

The classical psychedelics discussed in this paper have been shown to exert strong anti-cancer and anti-inflammatory effects through the modulation of innate and adaptive immune processes. The molecular biological background of these effects has not been investigated so far. Two models are proposed here to cover the possible biochemical dynamics of these interactions.

On the one hand, (i) regulation may occur through the alteration of the cytokine-pattern of activated cells. The anti-inflammatory cytokines, IL-10 and TGFβ, and pro-inflammatory cytokines, TNFα and IFNγ, seem to be key players in this regulation (Figure 2) (73–75). On the other hand, (ii) a complex intracellular cross-talk of PRRs, serotonin, and sigma-1 receptors might be involved in the immunomodulatory process. This may happen via the 5-HTR/sigmar-1-mediated modulation of intracellular Ca^2+^ levels and the activity of MAPKs and NF-κB, common components of signaling pathways highly involved in cellular proliferation, survival, and inflammation (Figure 1). Furthermore, interacting PRR and 5-HTR/sigmar-1 pathways may compete for common elements of downstream signaling (e.g., kinases, adaptor proteins), a phenomenon that can also lead to a significant inhibition of one of the interacting partners. A similar mechanism may lead to the preference of a given pathway through kinase or receptor–adaptor bias (145). An interesting contemporary approach to the topic has been carried out by using systems biology, bioinformatics, and biophysics, as tools of better understanding. This approach emphasizes that instead of single cell analyses, one should move toward a more holistic understanding of signaling systems. The meta-network of biological entities is considered to possess both microscopic and macroscopic dynamics as observed in physical sciences. The origin of averaging effects from stochastic responses of a single cell when collected to form a population should also be taken into account (146, 147). It is very likely that the emergence of an average cell deterministic response (e.g., following a PRR and/or 5-HTR stimulus) from single cell stochastic responses complements each other (20, 148, 149). Consequently, the stochastic fluctuations in the inflammatory response of a single immune cell or a single signaling pathway are necessary to induce probabilistic differentiation from identical cells or interacting pathways of the same receptor family. This might allow multicellular organisms or complex, interacting signaling networks to switch cell fates or states to yield diversity, fine-tuning, and reach the proper response that cannot be achieved by a purely deterministic system. Recent studies of multi-component, non-linear modeling of different TLR pathways verified the success of this idea by identifying cross-talk mechanisms between the MyD88- and TRIF-dependent pathways and led to the concept of signaling flux redistribution (SFR) (149, 150). This proposal is based on the law of conservation where the removal of MyD88 leads to increased activation of the entire alternative TRIF-pathway. Thus, total signaling flux information from a receptor through final downstream gene activation in the network is conserved. The group experimentally validated the SFR theory by using MyD88^−/−^ and TRAF6^−/−^ KO mice and their data generated interesting interpretations (150), which may open up new aspects toward the deeper understanding of cellular signaling processes (20). An important limitation of this double-model hypothesis is that available experimental data supporting the proposed interactions is mostly scarce. Thus, further studies are needed to confirm its relevance in future immunopharmacotherapies, especially as far as translational aspects and human clinical trials are concerned.

While PRRs were shown to be crucial for innate and adaptive host defense, their inappropriate activation has been associated with autoimmunity and inflammatory diseases. Psychedelics, by modulating the activity of 5-HT_1_, 5-HT_2_, and sigmar-1 receptors, are potent anti-inflammatory agents (70, 104, 128, 130–134). A more complete appreciation of the PRR-5-HTR/sigmar-1 cross-talk and their complex signaling processes would provide important insights into new therapeutic modalities that can either enhance immune responses or inhibit functions to diminish the deleterious effects of uncontrolled inflammation. Thus, these compounds emerge as very promising candidates in many diseases with chronic inflammatory etiology and pathology, such as atherosclerosis, psoriasis, rheumatoid arthritis, systemic lupus erythematosus, type I diabetes, multiple sclerosis, schizophrenia, depression, and Alzheimer’s disease.

## Conflict of Interest Statement

The author declares that the research was conducted in the absence of any commercial or financial relationships that could be construed as a potential conflict of interest.

## References

[1] Aghajanian GK Marek GJ Serotonin and hallucinogens. Neuropsychopharmacology (1999) 21(2 Suppl):16S–23S. 10.1016/S0893-133X(98)00135-3 10432484

[2] Greyer M Nichols D F. V. Serotonin-related psychedelic drugs. In: Squire L, editor. Encyclopedia of Neuroscience. Oxford: Academic Press (2009). p. 741–8.

[3] Baumeister D Barnes G Giaroli G Tracy D. Classical hallucinogens as antidepressants? A review of pharmacodynamics and putative clinical roles. Ther Adv Psychopharmacol (2014) 4(4):156–69. 10.1177/2045125314527985 25083275 PMC4104707

[4] Frecska E Luna LE. The adverse effects of hallucinogens from intramural perspective. Neuropsychopharmacol Hung (2006) 8(4):189–200. 17211054

[5] Quan N Banks WA. Brain-immune communication pathways. Brain Behav Immun (2007) 21(6):727–35. 10.1016/j.bbi.2007.05.005 17604598

[6] Szabo A Rajnavolgyi E The brain-immune-gut triangle: innate immunity in psychiatric and neurological disorders. Curr Immunol Rev (2013) 9:241–8. 10.2174/1573395509666131203225659

[7] Luna LE. The healing practices of a Peruvian shaman. J Ethnopharmacol (1984) 11(2):123–33. 10.1016/0378-8741(84)90035-7 6387284

[8] Winkelman M. A cross-cultural study of shamanistic healers. J Psychoactive Drugs (1989) 21(1):17–24. 10.1080/02791072.1989.10472139 2656949

[9] Moriarty KM Alagna SW Lake CR. Psychopharmacology. An historical perspective. Psychiatr Clin North Am (1984) 7(3):411–33. 6384957

[10] Alper KR Ibogaine: a review. Alkaloids Chem Biol (2001) 56:1–38. 10.1016/S0099-9598(01)56005-8 11705103

[11] Prue R Voss RW. Indigenous healing practice: ayahuasca. Opening a discussion. J Pastoral Care Counsel (2014) 68(1–2):6. 25241484

[12] Felten DL Neurotransmitter signaling of cells of the immune system: important progress, major gaps. Brain Behav Immun (1991) 5(1):2–8. 10.1016/0889-1591(91)90003-S 1648984

[13] Mossner R Lesch KP. Role of serotonin in the immune system and in neuroimmune interactions. Brain Behav Immun (1998) 12(4):249–71. 10.1006/brbi.1998.0532 10080856

[14] Sarkar C Basu B Chakroborty D Dasgupta PS Basu S. The immunoregulatory role of dopamine: an update. Brain Behav Immun (2010) 24(4):525–8. 10.1016/j.bbi.2009.10.015 19896530 PMC2856781

[15] de Las Casas-Engel M Corbi AL. Serotonin modulation of macrophage polarization: inflammation and beyond. Adv Exp Med Biol (2014) 824:89–115. 10.1007/978-3-319-07320-0_9 25038996

[16] Gordon J Barnes NM Lymphocytes transport serotonin and dopamine: agony or ecstasy? Trends Immunol (2003) 24(8):438–43. 10.1016/S1471-4906(03)00176-5 12909457

[17] Raymond JR Mukhin YV Gelasco A Turner J Collinsworth G Gettys TW Multiplicity of mechanisms of serotonin receptor signal transduction. Pharmacol Ther (2001) 92(2–3):179–212. 10.1016/S0163-7258(01)00169-3 11916537

[18] Mukhin YV Garnovskaya MN Collinsworth G Grewal JS Pendergrass D Nagai T 5-Hydroxytryptamine1A receptor/Gibetagamma stimulates mitogen-activated protein kinase via NAD(P)H oxidase and reactive oxygen species upstream of src in Chinese hamster ovary fibroblasts. Biochem J (2000) 347(Pt 1):61–7. 10.1042/0264-6021:3470061 10727402 PMC1220931

[19] Cowen DS Molinoff PB Manning DR. 5-hydroxytryptamine1A receptor-mediated increases in receptor expression and activation of nuclear factor-kappaB in transfected Chinese hamster ovary cells. Mol Pharmacol (1997) 52(2):221–6. 9271344 10.1124/mol.52.2.221

[20] Szabo A Rajnavolgyi E. Collaboration of toll-like and RIG-I-like receptors in human dendritic cells: tRIGgering antiviral innate immune responses. Am J Clin Exp Immunol (2013) 2(3):195–207. 24179728 PMC3808934

[21] Grotewiel MS Sanders-Bush E. Differences in agonist-independent activity of 5-Ht2A and 5-HT2c receptors revealed by heterologous expression. Naunyn Schmiedebergs Arch Pharmacol (1999) 359(1):21–7. 10.1007/PL00005318 9933146

[22] Goppelt-Struebe M Hahn A Stroebel M Reiser CO. Independent regulation of cyclo-oxygenase 2 expression by p42/44 mitogen-activated protein kinases and Ca2+/calmodulin-dependent kinase. Biochem J (1999) 339(Pt 2):329–34. 10.1042/0264-6021:3390329 10191263 PMC1220161

[23] Grewal JS Mukhin YV Garnovskaya MN Raymond JR Greene EL. Serotonin 5-HT2A receptor induces TGF-beta1 expression in mesangial cells via ERK: proliferative and fibrotic signals. Am J Physiol (1999) 276(6 Pt 2):F922–30. 10362781 10.1152/ajprenal.1999.276.6.F922

[24] Guillet-Deniau I Burnol AF Girard J. Identification and localization of a skeletal muscle serotonin 5-HT2A receptor coupled to the Jak/STAT pathway. J Biol Chem (1997) 272(23):14825–9. 10.1074/jbc.272.23.14825 9169451

[25] Barnes NM Sharp T A review of central 5-HT receptors and their function. Neuropharmacology (1999) 38(8):1083–152. 10.1016/S0028-3908(99)00010-6 10462127

[26] Launay JM Birraux G Bondoux D Callebert J Choi DS Loric S Ras involvement in signal transduction by the serotonin 5-HT2B receptor. J Biol Chem (1996) 271(6):3141–7. 10.1074/jbc.271.6.3141 8621713

[27] Idzko M Panther E Stratz C Muller T Bayer H Zissel G The serotoninergic receptors of human dendritic cells: identification and coupling to cytokine release. J Immunol (2004) 172(10):6011–9. 10.4049/jimmunol.172.10.6011 15128784

[28] Wolf WA Schutz LJ. The serotonin 5-HT2C receptor is a prominent serotonin receptor in basal ganglia: evidence from functional studies on serotonin-mediated phosphoinositide hydrolysis. J Neurochem (1997) 69(4):1449–58. 10.1046/j.1471-4159.1997.69041449.x 9326273

[29] Mikulski Z Zaslona Z Cakarova L Hartmann P Wilhelm J Tecott LH Serotonin activates murine alveolar macrophages through 5-HT2C receptors. Am J Physiol Lung Cell Mol Physiol (2010) 299(2):L272–80. 10.1152/ajplung.00032.2010 20495077

[30] Stefulj J Jernej B Cicin-Sain L Rinner I Schauenstein K. mRNA expression of serotonin receptors in cells of the immune tissues of the rat. Brain Behav Immun (2000) 14(3):219–24. 10.1006/brbi.1999.0579 10970681

[31] Assetta B Maginnis MS Gracia Ahufinger I Haley SA Gee GV Nelson CD 5-HT2 receptors facilitate JC polyomavirus entry. J Virol (2013) 87(24):13490–8. 10.1128/JVI.02252-13 24089568 PMC3838264

[32] Aune TM McGrath KM Sarr T Bombara MP Kelley KA. Expression of 5HT1a receptors on activated human T cells. Regulation of cyclic AMP levels and T cell proliferation by 5-hydroxytryptamine. J Immunol (1993) 151(3):1175–83. 8393041

[33] Eugen-Olsen J Afzelius P Andresen L Iversen J Kronborg G Aabech P Serotonin modulates immune function in T cells from HIV-seropositive subjects. Clin Immunol Immunopathol (1997) 84(2):115–21. 10.1006/clin.1997.4384 9245541

[34] Miller AH. Norman cousins lecture. Mechanisms of cytokine-induced behavioral changes: psychoneuroimmunology at the translational interface. Brain Behav Immun (2009) 23(2):149–58. 10.1016/j.bbi.2008.08.006 18793712 PMC2745948

[35] Guo AL Petraglia F Criscuolo M Ficarra G Salvestroni C Nappi RE Adrenergic and serotoninergic receptors mediate the immunological activation of corticosterone secretion in male rats. Gynecol Endocrinol (1996) 10(3):149–54. 10.3109/09513599609027981 8862488

[36] Leonard BE. The HPA and immune axes in stress: the involvement of the serotonergic system. Eur Psychiatry (2005) 20(Suppl 3):S302–6. 10.1016/S0924-9338(05)80180-4 16459240

[37] Su TP Hayashi T Maurice T Buch S Ruoho AE. The sigma-1 receptor chaperone as an inter-organelle signaling modulator. Trends Pharmacol Sci (2010) 31(12):557–66. 10.1016/j.tips.2010.08.007 20869780 PMC2993063

[38] Pabba M The essential roles of protein-protein interaction in sigma-1 receptor functions. Front Cell Neurosci (2013) 7:50 10.3389/fncel.2013.00050 23630466 PMC3633076

[39] Kourrich S Su TP Fujimoto M Bonci A. The sigma-1 receptor: roles in neuronal plasticity and disease. Trends Neurosci (2012) 35(12):762–71. 10.1016/j.tins.2012.09.007 23102998 PMC3587126

[40] Zhang H Cuevas J. Sigma receptors inhibit high-voltage-activated calcium channels in rat sympathetic and parasympathetic neurons. J Neurophysiol (2002) 87(6):2867–79. 12037190 10.1152/jn.2002.87.6.2867

[41] Fontanilla D Johannessen M Hajipour AR Cozzi NV Jackson MB Ruoho AE. The hallucinogen N,N-dimethyltryptamine (DMT) is an endogenous sigma-1 receptor regulator. Science (2009) 323(5916):934–7. 10.1126/science.1166127 19213917 PMC2947205

[42] Su TP Hayashi T Vaupel DB. When the endogenous hallucinogenic trace amine N,N-dimethyltryptamine meets the sigma-1 receptor. Sci Signal (2009) 2(61):e12. 10.1126/scisignal.261pe12 19278957 PMC3155724

[43] Hayashi T Rizzuto R Hajnoczky G Su TP. MAM: more than just a housekeeper. Trends Cell Biol (2009) 19(2):81–8. 10.1016/j.tcb.2008.12.002 19144519 PMC2750097

[44] Hayashi T Su TP. Intracellular dynamics of sigma-1 receptors (sigma(1) binding sites) in NG108-15 cells. J Pharmacol Exp Ther (2003) 306(2):726–33. 10.1124/jpet.103.051292 12730356

[45] Mavlyutov TA Ruoho AE. Ligand-dependent localization and intracellular stability of sigma-1 receptors in CHO-K1 cells. J Mol Signal (2007) 2:8. 10.1186/1750-2187-2-8 17883859 PMC2045653

[46] Aydar E Palmer CP Klyachko VA Jackson MB. The sigma receptor as a ligand-regulated auxiliary potassium channel subunit. Neuron (2002) 34(3):399–410. 10.1016/S0896-6273(02)00677-3 11988171

[47] Su TP London ED Jaffe JH. Steroid binding at sigma receptors suggests a link between endocrine, nervous, and immune systems. Science (1988) 240(4849):219–21. 10.1126/science.2832949 2832949

[48] Hellewell SB Bruce A Feinstein G Orringer J Williams W Bowen WD. Rat liver and kidney contain high densities of sigma 1 and sigma 2 receptors: characterization by ligand binding and photoaffinity labeling. Eur J Pharmacol (1994) 268(1):9–18. 10.1016/0922-4106(94)90115-5 7925616

[49] Wolfe SA Jr Kulsakdinun C Battaglia G Jaffe JH De Souza EB. Initial identification and characterization of sigma receptors on human peripheral blood leukocytes. J Pharmacol Exp Ther (1988) 247(3):1114–9. 2849660

[50] Zhu LX Sharma S Gardner B Escuadro B Atianzar K Tashkin DP IL-10 mediates sigma 1 receptor-dependent suppression of antitumor immunity. J Immunol (2003) 170(7):3585–91. 10.4049/jimmunol.170.7.3585 12646621

[51] Carayon P Bouaboula M Loubet JF Bourrie B Petitpretre G Le Fur G The sigma ligand SR 31747 prevents the development of acute graft-versus-host disease in mice by blocking IFN-gamma and GM-CSF mRNA expression. Int J Immunopharmacol (1995) 17(9):753–61. 10.1016/0192-0561(95)00066-B 8582787

[52] Bourrie B Bouaboula M Benoit JM Derocq JM Esclangon M Le Fur G Enhancement of endotoxin-induced interleukin-10 production by SR 31747A, a sigma ligand. Eur J Immunol (1995) 25(10):2882–7. 10.1002/eji.1830251026 7589087

[53] Nunez G Intracellular sensors of microbes and danger. Immunol Rev (2011) 243(1):5–8. 10.1111/j.1600-065X.2011.01058.x 21884163

[54] Bowie AG Unterholzner L. Viral evasion and subversion of pattern-recognition receptor signalling. Nat Rev Immunol (2008) 8(12):911–22. 10.1038/nri2436 18989317 PMC7097711

[55] Kawai T Akira S Antiviral signaling through pattern recognition receptors. J Biochem (2007) 141(2):137–45. 10.1093/jb/mvm032 17190786

[56] Rock KL Lai JJ Kono H Innate and adaptive immune responses to cell death. Immunol Rev (2011) 243(1):191–205. 10.1111/j.1600-065X.2011.01040.x 21884177 PMC3170128

[57] Barber GN. Innate immune DNA sensing pathways: STING, AIMII and the regulation of interferon production and inflammatory responses. Curr Opin Immunol (2011) 23(1):10–20. 10.1016/j.coi.2010.12.015 21239155 PMC3881186

[58] Olive C. Pattern recognition receptors: sentinels in innate immunity and targets of new vaccine adjuvants. Expert Rev Vaccines (2012) 11(2):237–56. 10.1586/erv.11.189 22309671

[59] Barbalat R Ewald SE Mouchess ML Barton GM. Nucleic acid recognition by the innate immune system. Annu Rev Immunol (2011) 29:185–214. 10.1146/annurev-immunol-031210-101340 21219183

[60] Kawai T Akira S. Toll-like receptor and RIG-I-like receptor signaling. Ann N Y Acad Sci (2008) 1143:1–20. 10.1196/annals.1443.020 19076341

[61] Benko S Magyarics Z Szabo A Rajnavolgyi E. Dendritic cell subtypes as primary targets of vaccines: the emerging role and cross-talk of pattern recognition receptors. Biol Chem (2008) 389(5):469–85. 10.1515/BC.2008.054 18953714

[62] Blander JM. A long-awaited merger of the pathways mediating host defence and programmed cell death. Nat Rev Immunol (2014) 14(9):601–18. 10.1038/nri3720 25145756

[63] Chen H Jiang Z. The essential adaptors of innate immune signaling. Protein Cell (2013) 4(1):27–39. 10.1007/s13238-012-2063-0 22996173 PMC4875439

[64] Unterholzner L Bowie AG. The interplay between viruses and innate immune signaling: recent insights and therapeutic opportunities. Biochem Pharmacol (2008) 75(3):589–602. 10.1016/j.bcp.2007.07.043 17868652

[65] Szabo A Rajnavolgyi E Finding a fairy in the forest: ELF4, a novel and critical element of type I interferon responses. Cell Mol Immunol (2014) 11(3):218–20. 10.1038/cmi.2014.1 24658434 PMC4085491

[66] Lee MS Kim YJ. Signaling pathways downstream of pattern-recognition receptors and their cross talk. Annu Rev Biochem (2007) 76:447–80. 10.1146/annurev.biochem.76.060605.122847 17328678

[67] Doria A Zen M Bettio S Gatto M Bassi N Nalotto L Autoinflammation and autoimmunity: bridging the divide. Autoimmun Rev (2012) 12(1):22–30. 10.1016/j.autrev.2012.07.018 22878274

[68] Rogers TJ. The molecular basis for neuroimmune receptor signaling. J Neuroimmune Pharmacol (2012) 7(4):722–4. 10.1007/s11481-012-9398-4 22935971 PMC4011130

[69] Cloez-Tayarani I Changeux JP. Nicotine and serotonin in immune regulation and inflammatory processes: a perspective. J Leukoc Biol (2007) 81(3):599–606. 10.1189/jlb.0906544 17108054

[70] Cloez-Tayarani I Petit-Bertron AF Venters HD Cavaillon JM. Differential effect of serotonin on cytokine production in lipopolysaccharide-stimulated human peripheral blood mononuclear cells: involvement of 5-hydroxytryptamine2A receptors. Int Immunol (2003) 15(2):233–40. 10.1093/intimm/dxg027 12578853

[71] Durk T Panther E Muller T Sorichter S Ferrari D Pizzirani C 5-Hydroxytryptamine modulates cytokine and chemokine production in LPS-primed human monocytes via stimulation of different 5-HTR subtypes. Int Immunol (2005) 17(5):599–606. 10.1093/intimm/dxh242 15802305

[72] Foon KA Wahl SM Oppenheim JJ Rosenstreich DL. Serotonin-induced production of a monocyte chemotactic factor by human peripheral blood leukocytes. J Immunol (1976) 117(5 Pt 1):1545–52. 1002988

[73] Kubera M Kenis G Bosmans E Scharpe S Maes M. Effects of serotonin and serotonergic agonists and antagonists on the production of interferon-gamma and interleukin-10. Neuropsychopharmacology (2000) 23(1):89–98. 10.1016/S0893-133X(99)00150-5 10869889

[74] Maes M Kenis G Bosmans E. The negative immunoregulatory effects of serotonin (5-HT) moduline, an endogenous 5-HT1B receptor antagonist with anti-anxiety properties. Cytokine (2002) 19(6):308–11. 10.1006/cyto.2002.1053 12421573

[75] de las Casas-Engel M Dominguez-Soto A Sierra-Filardi E Bragado R Nieto C Puig-Kroger A Serotonin skews human macrophage polarization through HTR2B and HTR7. J Immunol (2013) 190(5):2301–10. 10.4049/jimmunol.1201133 23355731

[76] Ye RD. Regulation of nuclear factor kappaB activation by G-protein-coupled receptors. J Leukoc Biol (2001) 70(6):839–48. 11739545

[77] Wang D Richmond A. Nuclear factor-kappa B activation by the CXC chemokine melanoma growth-stimulatory activity/growth-regulated protein involves the MEKK1/p38 mitogen-activated protein kinase pathway. J Biol Chem (2001) 276(5):3650–9. 10.1074/jbc.M006115200 11062239 PMC2676351

[78] Ito T Ikeda U Shimpo M Yamamoto K Shimada K. Serotonin increases interleukin-6 synthesis in human vascular smooth muscle cells. Circulation (2000) 102(20):2522–7. 10.1161/01.CIR.102.20.2522 11076827

[79] Abdouh M Albert PR Drobetsky E Filep JG Kouassi E. 5-HT1A-mediated promotion of mitogen-activated T and B cell survival and proliferation is associated with increased translocation of NF-kappaB to the nucleus. Brain Behav Immun (2004) 18(1):24–34. 10.1016/S0889-1591(03)00088-6 14651944

[80] Hsiung SC Tamir H Franke TF Liu KP. Roles of extracellular signal-regulated kinase and Akt signaling in coordinating nuclear transcription factor-kappaB-dependent cell survival after serotonin 1A receptor activation. J Neurochem (2005) 95(6):1653–66. 10.1111/j.1471-4159.2005.03496.x 16238696

[81] Soga F Katoh N Inoue T Kishimoto S. Serotonin activates human monocytes and prevents apoptosis. J Invest Dermatol (2007) 127(8):1947–55. 10.1038/sj.jid.5700824 17429435

[82] Xie P Browning DD Hay N Mackman N Ye RD. Activation of NF-kappa B by bradykinin through a Galpha(q)- and Gbeta gamma-dependent pathway that involves phosphoinositide 3-kinase and Akt. J Biol Chem (2000) 275(32):24907–14. 10.1074/jbc.M001051200 10801799

[83] Gutkind JS The pathways connecting G protein-coupled receptors to the nucleus through divergent mitogen-activated protein kinase cascades. J Biol Chem (1998) 273(4):1839–42. 10.1074/jbc.273.4.1839 9442012

[84] Hedin KE Bell MP Huntoon CJ Karnitz LM McKean DJ. Gi proteins use a novel beta gamma- and Ras-independent pathway to activate extracellular signal-regulated kinase and mobilize AP-1 transcription factors in Jurkat T lymphocytes. J Biol Chem (1999) 274(28):19992–20001. 10.1074/jbc.274.28.19992 10391949

[85] Fraser CC. G protein-coupled receptor connectivity to NF-kappaB in inflammation and cancer. Int Rev Immunol (2008) 27(5):320–50. 10.1080/08830180802262765 18853342

[86] Jiang HY Deng M Zhang YH Chen HZ Chen Q Ruan B. Specific serotonin reuptake inhibitors prevent interferon-alpha-induced depression in patients with hepatitis C: a meta-analysis. Clin Gastroenterol Hepatol (2014) 12(9):1452–60e3. 10.1016/j.cgh.2013.04.035 23648373

[87] Ehret M Sobieraj DM. Prevention of interferon-alpha-associated depression with antidepressant medications in patients with hepatitis C virus: a systematic review and meta-analysis. Int J Clin Pract (2014) 68(2):255–61. 10.1111/ijcp.12268 24372654

[88] Tourino MC de Oliveira EM Belle LP Knebel FH Albuquerque RC Dorr FA Tryptamine and dimethyltryptamine inhibit indoleamine 2,3 dioxygenase and increase the tumor-reactive effect of peripheral blood mononuclear cells. Cell Biochem Funct (2013) 31(5):361–4. 10.1002/cbf.2980 23754498

[89] Schenberg E Ayahuasca and cancer treatment. SAGE Open Med (2013) 1 10.1177/2050312113508389 PMC4687784 26770688

[90] House RV Thomas PT Bhargava HN. Immunological consequences of in vitro exposure to lysergic acid diethylamide (LSD). Immunopharmacol Immunotoxicol (1994) 16(1):23–40. 10.3109/08923979409029898 8169321

[91] Barker SA McIlhenny EH Strassman R. A critical review of reports of endogenous psychedelic N, N-dimethyltryptamines in humans: 1955-2010. Drug Test Anal (2012) 4(7–8):617–35. 10.1002/dta.422 22371425

[92] Luna E Indigenous and mestizo use of ayahuasca: an overview. In: Dos Santos RG, editor. The Ethnopharmacology of Ayahuasca. Kerala: Transworld Research Network (2011). p. 1–22.

[93] Wallach JV. Endogenous hallucinogens as ligands of the trace amine receptors: a possible role in sensory perception. Med Hypotheses (2009) 72(1):91–4. 10.1016/j.mehy.2008.07.052 18805646

[94] Szara S Dimethyltryptamin: its metabolism in man; the relation to its psychotic effect to the serotonin metabolism. Experientia (1956) 12(11):441–2. 10.1007/BF02157378 13384414

[95] Axelrod J. Enzymatic formation of psychotomimetic metabolites from normally occurring compounds. Science (1961) 134(3475):343. 10.1126/science.134.3475.343 13685339

[96] Christian ST Harrison R Quayle E Pagel J Monti J The in vitro identification of dimethyltryptamine (DMT) in mammalian brain and its characterization as a possible endogenous neuroregulatory agent. Biochem Med (1977) 18(2):164–83. 10.1016/0006-2944(77)90088-6 20877

[97] Holister L Some general thoughts about endogenous psychotogens. In: Usdin E Hamburg DA Barchas JD, editors. Neuroregulators and Psychiatric Disorders. New York, NY: Oxford University Press (1977). p. 550–6.

[98] Barker SA Monti JA Christian ST N, N-dimethyltryptamine: an endogenous hallucinogen. Int Rev Neurobiol (1981) 22:83–110. 10.1016/S0074-7742(08)60291-3 6792104

[99] Deliganis AV Pierce PA Peroutka SJ. Differential interactions of dimethyltryptamine (DMT) with 5-HT1A and 5-HT2 receptors. Biochem Pharmacol (1991) 41(11):1739–44. 10.1016/0006-2952(91)90178-8 1828347

[100] Keiser MJ Setola V Irwin JJ Laggner C Abbas AI Hufeisen SJ Predicting new molecular targets for known drugs. Nature (2009) 462(7270):175–81. 10.1038/nature08506 19881490 PMC2784146

[101] Ray TS. Psychedelics and the human receptorome. PLoS One (2010) 5(2):e9019. 10.1371/journal.pone.0009019 20126400 PMC2814854

[102] Smith RL Canton H Barrett RJ Sanders-Bush E. Agonist properties of N,N-dimethyltryptamine at serotonin 5-HT2A and 5-HT2C receptors. Pharmacol Biochem Behav (1998) 61(3):323–30. 10.1016/S0091-3057(98)00110-5 9768567

[103] Frecska E Szabo A Winkelman MJ Luna LE McKenna DJ. A possibly sigma-1 receptor mediated role of dimethyltryptamine in tissue protection, regeneration, and immunity. J Neural Transm (2013) 120(9):1295–303. 10.1007/s00702-013-1024-y 23619992

[104] Szabo A Kovacs A Frecska E Rajnavolgyi E. Psychedelic N,N-dimethyltryptamine and 5-methoxy-N,N-dimethyltryptamine modulate innate and adaptive inflammatory responses through the sigma-1 receptor of human monocyte-derived dendritic cells. PLoS One (2014) 9(8):e106533. 10.1371/journal.pone.0106533 25171370 PMC4149582

[105] Dos Santos RG Valle M Bouso JC Nomdedeu JF Rodriguez-Espinosa J McIlhenny EH Autonomic, neuroendocrine, and immunological effects of ayahuasca: a comparative study with d-amphetamine. J Clin Psychopharmacol (2011) 31(6):717–26. 10.1097/JCP.0b013e31823607f6 22005052

[106] Dos Santos RG Grasa E Valle M Ballester MR Bouso JC Nomdedeu JF Pharmacology of ayahuasca administered in two repeated doses. Psychopharmacology (2012) 219(4):1039–53. 10.1007/s00213-011-2434-x 21842159

[107] Dos Santos RG. Immunological effects of ayahuasca in humans. J Psychoactive Drugs (2014) 46(5):383–8. 10.1080/02791072.2014.960113 25364989

[108] Zhu J Yamane H Paul WE. Differentiation of effector CD4 T cell populations (*). Annu Rev Immunol (2010) 28:445–89. 10.1146/annurev-immunol-030409-101212 20192806 PMC3502616

[109] Pierson E Simmons SB Castelli L Goverman JM. Mechanisms regulating regional localization of inflammation during CNS autoimmunity. Immunol Rev (2012) 248(1):205–15. 10.1111/j.1600-065X.2012.01126.x 22725963 PMC3678350

[110] Passie T Halpern JH Stichtenoth DO Emrich HM Hintzen A. The pharmacology of lysergic acid diethylamide: a review. CNS Neurosci Ther (2008) 14(4):295–314. 10.1111/j.1755-5949.2008.00059.x 19040555 PMC6494066

[111] Aghajanian GK Bing OH Persistence of lysergic acid diethylamide in the plasma of human subjects. Clin Pharmacol Ther (1964) 5:611–4. 14209776 10.1002/cpt196455611

[112] Urban JD Clarke WP von Zastrow M Nichols DE Kobilka B Weinstein H Functional selectivity and classical concepts of quantitative pharmacology. J Pharmacol Exp Ther (2007) 320(1):1–13. 10.1124/jpet.106.104463 16803859

[113] Voss EW Jr Winkelhake JL. Mechanism of lysergic acid diethylamide interference with rabbit antibody biosynthesis. Proc Natl Acad Sci U S A (1974) 71(4):1061–4. 10.1073/pnas.71.4.1061 4524614 PMC388162

[114] Fukuda Y Koga M Arai M Noguchi E Ohtsuki T Horiuchi Y Monoallelic and unequal allelic expression of the HTR2A gene in human brain and peripheral lymphocytes. Biol Psychiatry (2006) 60(12):1331–5. 10.1016/j.biopsych.2006.06.024 17069769

[115] Martins LC Rocha NP Torres KC Dos Santos RR Franca GS de Moraes EN Disease-specific expression of the serotonin-receptor 5-HT(2C) in natural killer cells in Alzheimer’s dementia. J Neuroimmunol (2012) 251(1–2):73–9. 10.1016/j.jneuroim.2012.06.003 22766135

[116] Hagel JM Krizevski R Marsolais F Lewinsohn E Facchini PJ. Biosynthesis of amphetamine analogs in plants. Trends Plant Sci (2012) 17(7):404–12. 10.1016/j.tplants.2012.03.004 22502775

[117] Wood S Sage JR Shuman T Anagnostaras SG. Psychostimulants and cognition: a continuum of behavioral and cognitive activation. Pharmacol Rev (2014) 66(1):193–221. 10.1124/pr.112.007054 24344115 PMC3880463

[118] Dean BV Stellpflug SJ Burnett AM Engebretsen KM. 2C or not 2C: phenethylamine designer drug review. J Med Toxicol (2013) 9(2):172–8. 10.1007/s13181-013-0295-x 23494844 PMC3657019

[119] Canal CE Morgan D. Head-twitch response in rodents induced by the hallucinogen 2,5-dimethoxy-4-iodoamphetamine: a comprehensive history, a re-evaluation of mechanisms, and its utility as a model. Drug Test Anal (2012) 4(7–8):556–76. 10.1002/dta.1333 22517680 PMC3722587

[120] Moreno JL Holloway T Albizu L Sealfon SC Gonzalez-Maeso J. Metabo-tropic glutamate mGlu2 receptor is necessary for the pharmacological and behavioral effects induced by hallucinogenic 5-HT2A receptor agonists. Neurosci Lett (2011) 493(3):76–9. 10.1016/j.neulet.2011.01.046 21276828 PMC3064746

[121] Lyon RA Glennon RA Titeler M. 3,4-Methylenedioxymethamphetamine (MDMA): stereoselective interactions at brain 5-HT1 and 5-HT2 receptors. Psychopharmacology (1986) 88(4):525–6. 10.1007/BF00178519 2871581

[122] Battaglia G Brooks BP Kulsakdinun C De Souza EB. Pharmacologic profile of MDMA (3,4-methylenedioxymethamphetamine) at various brain recognition sites. Eur J Pharmacol (1988) 149(1–2):159–63. 10.1016/0014-2999(88)90056-8 2899513

[123] Setola V Hufeisen SJ Grande-Allen KJ Vesely I Glennon RA Blough B 3,4-methylenedioxymethamphetamine (MDMA, “Ecstasy”) induces fenfluramine-like proliferative actions on human cardiac valvular interstitial cells in vitro. Mol Pharmacol (2003) 63(6):1223–9. 10.1124/mol.63.6.1223 12761331

[124] McKenna DJ Mathis CA Shulgin AT Sargent T III Saavedra JM Autoradiographic localization of binding sites for 125I-DOI, a new psychotomimetic radioligand, in the rat brain. Eur J Pharmacol (1987) 137(2–3):289–90. 10.1016/0014-2999(87)90239-1 3609149

[125] Nazarali AJ McKenna DJ Saavedra JM. Autoradiographic localization of 5HT2 receptors in rat brain using [125I]-DOI, a selective psychotomimetic radioligand. Prog Neuropsychopharmacol Biol Psychiatry (1989) 13(3–4):573–81. 10.1016/0278-5846(89)90149-8 2748882

[126] Miller KJ Mariano CL Cruz WR. Serotonin 5HT2A receptor activation inhibits inducible nitric oxide synthase activity in C6 glioma cells. Life Sci (1997) 61(18):1819–27. 10.1016/S0024-3205(97)00806-0 9365229

[127] Miller KJ Gonzalez HA. Serotonin 5-HT2A receptor activation inhibits cytokine-stimulated inducible nitric oxide synthase in C6 glioma cells. Ann N Y Acad Sci (1998) 861:169–73. 10.1111/j.1749-6632.1998.tb10188.x 9928254

[128] Yu B Becnel J Zerfaoui M Rohatgi R Boulares AH Nichols CD. Serotonin 5-hydroxytryptamine(2A) receptor activation suppresses tumor necrosis factor-alpha-induced inflammation with extraordinary potency. J Pharmacol Exp Ther (2008) 327(2):316–23. 10.1124/jpet.108.143461 18708586

[129] Nau F Jr Miller J Saravia J Ahlert T Yu B Happel KI Serotonin 5-HT2 receptor activation prevents allergic asthma in a mouse model. Am J Physiol Lung Cell Mol Physiol (2014) 308(2):L191–8. 10.1152/ajplung.00138.2013 25416380 PMC4338939

[130] House RV Thomas PT Bhargava HN. Selective modulation of immune function resulting from in vitro exposure to methylenedioxymethamphetamine (Ecstasy). Toxicology (1995) 96(1):59–69. 10.1016/0300-483X(94)02955-T 7863512

[131] Connor TJ McNamara MG Finn D Currid A O’Malley M Redmond AM Acute 3,4-methylenedioxymethamphetamine(MDMA) administration produces a rapid and sustained suppression of immune function in the rat. Immunopharmacology (1998) 38(3):253–60. 10.1016/S0162-3109(97)00084-2 9506825

[132] Connor TJ Kelly JP McGee M Leonard BE. Methylenedioxymethamphetamine (MDMA; Ecstasy) suppresses IL-1beta and TNF-alpha secretion following an in vivo lipopolysaccharide challenge. Life Sci (2000) 67(13):1601–12. 10.1016/S0024-3205(00)00743-8 10983854

[133] Connor TJ Kelly JP Leonard BE. An assessment of the acute effects of the serotonin releasers methylenedioxymethamphetamine, methylenedioxyamphetamine and fenfluramine on immunity in rats. Immunopharmacology (2000) 46(3):223–35. 10.1016/S0162-3109(99)00180-0 10741902

[134] Boyle NT Connor TJ. Methylenedioxymethamphetamine (‘Ecstasy’)-induced immunosuppression: a cause for concern? Br J Pharmacol (2010) 161(1):17–32. 10.1111/j.1476-5381.2010.00899.x 20718737 PMC2962814

[135] Connor TJ Connelly DB Kelly JP. Methylenedioxymethamphetamine (MDMA; ‘Ecstasy’) suppresses antigen specific IgG2a and IFN-gamma production. Immunol Lett (2001) 78(2):67–73. 10.1016/S0165-2478(01)00231-0 11672589

[136] Nelson DA Nirmaier JL Singh SJ Tolbert MD Bost KL. Ecstasy (3,4-methylenedioxymethamphetamine) limits murine gammaherpesvirus-68 induced monokine expression. Brain Behav Immun (2008) 22(6):912–22. 10.1016/j.bbi.2008.01.002 18280699 PMC4275657

[137] Pennock JW Stegall R Bubar MJ Milligan G Cunningham KA Bourne N. 3,4-Methylenedioxymethamphetamine increases susceptibility to genital herpes simplex virus infection in mice. J Infect Dis (2009) 200(8):1247–50. 10.1086/605891 19758099

[138] Connor TJ Harkin A Kelly JP. Methylenedioxymethamphetamine suppresses production of the proinflammatory cytokine tumor necrosis factor-alpha independent of a beta-adrenoceptor-mediated increase in interleukin-10. J Pharmacol Exp Ther (2005) 312(1):134–43. 10.1124/jpet.104.073023 15331655

[139] Camarasa J Ros C Pubill D Escubedo E. Tumour necrosis factor alpha suppression by MDMA is mediated by peripheral heteromeric nicotinic receptors. Immunopharmacol Immunotoxicol (2010) 32(2):265–71. 10.3109/08923970903295104 20105082

[140] Ferraz-de-Paula V Ribeiro A Souza-Queiroz J Pinheiro ML Vecina JF Souza DP 3,4-Methylenedioxymethamphetamine (MDMA – Ecstasy) decreases neutrophil activity through the glucocorticoid pathway and impairs host resistance to *Listeria* monocytogenes infection in mice. J Neuroimmune Pharmacol (2014) 9(5):690–702. 10.1007/s11481-014-9562-0 25113903

[141] Pacifici R Zuccaro P Farre M Pichini S Di Carlo S Roset PN Effects of repeated doses of MDMA (“ecstasy”) on cell-mediated immune response in humans. Life Sci (2001) 69(24):2931–41. 10.1016/S0024-3205(01)01373-X 11720096

[142] Pacifici R Zuccaro P Farre M Pichini S Di Carlo S Roset PN Cell-mediated immune response in MDMA users after repeated dose administration: studies in controlled versus noncontrolled settings. Ann N Y Acad Sci (2002) 965:421–33. 10.1111/j.1749-6632.2002.tb04183.x 12105117

[143] Pacifici R Pichini S Zuccaro P Farre M Segura M Ortuno J Paroxetine inhibits acute effects of 3,4-methylenedioxymethamphetamine on the immune system in humans. J Pharmacol Exp Ther (2004) 309(1):285–92. 10.1124/jpet.103.061374 14722327

[144] Pacifici R Zuccaro P Farre M Poudevida S Abanades S Pichini S Combined immunomodulating properties of 3,4-methylenedioxymethamphet amine (MDMA) and cannabis in humans. Addiction (2007) 102(6):931–6. 10.1111/j.1360-0443.2007.01805.x 17523988

[145] Kolb JP Casella CR SenGupta S Chilton PM Mitchell TC. Type I interferon signaling contributes to the bias that toll-like receptor 4 exhibits for signaling mediated by the adaptor protein TRIF. Sci Signal (2014) 7(351):ra108. 10.1126/scisignal.2005442 25389373 PMC4459894

[146] Zhao M Zhang J Phatnani H Scheu S Maniatis T. Stochastic expression of the interferon-beta gene. PLoS Biol (2012) 10(1):e1001249. 10.1371/journal.pbio.1001249 22291574 PMC3265471

[147] Hwang SY Hur KY Kim JR Cho KH Kim SH Yoo JY. Biphasic RLR-IFN-beta response controls the balance between antiviral immunity and cell damage. J Immunol (2013) 190(3):1192–200. 10.4049/jimmunol.1202326 23284052

[148] Gutierrez J St Laurent G III Urcuqui-Inchima S. Propagation of kinetic uncertainties through a canonical topology of the TLR4 signaling network in different regions of biochemical reaction space. Theor Biol Med Model (2010) 7:7. 10.1186/1742-4682-7-7 20230643 PMC2907738

[149] Selvarajoo K. Macroscopic law of conservation revealed in the population dynamics of toll-like receptor signaling. Cell Commun Signal (2011) 9:9. 10.1186/1478-811X-9-9 21507223 PMC3103489

[150] Selvarajoo K Takada Y Gohda J Helmy M Akira S Tomita M Signaling flux redistribution at toll-like receptor pathway junctions. PLoS One (2008) 3(10):e3430. 10.1371/journal.pone.0003430 18927610 PMC2561291

